# A retrospective ‘real-world’ cohort study of azole therapeutic drug monitoring and evolution of antifungal resistance in cystic fibrosis

**DOI:** 10.1093/jacamr/dlab026

**Published:** 2021-03-16

**Authors:** M Di Paolo, L Hewitt, E Nwankwo, M Ni, A Vidal-Diaz, M C Fisher, D Armstrong-James, A Shah

**Affiliations:** 1 Department of Respiratory Medicine, Royal Brompton and Harefield NHS Foundation Trust, London, UK; 2 Faculty of Medicine, Department of Infectious Diseases, Imperial College London, London, UK; 3 London In Vitro Diagnostics Collaborative, Department of Surgery and Cancer, Imperial College London, UK; 4 MRC Centre of Global Infectious Disease Analysis, Department of Infectious Disease Epidemiology, School of Public Health, Imperial College London, UK

## Abstract

**Background:**

Individuals with cystic fibrosis (CF) have an increased susceptibility to fungal infection/allergy, with triazoles often used as first-line therapy. Therapeutic drug monitoring (TDM) is essential due to significant pharmacokinetic variability and the recent emergence of triazole resistance worldwide.

**Objectives:**

In this retrospective study we analysed the ‘real-world’ TDM of azole therapy in a large CF cohort, risk factors for subtherapeutic dosing, and the emergence of azole resistance.

**Methods:**

All adults with CF on azole therapy in a large single UK centre were included. Clinical demographics, TDM and microbiology were analysed over a 2 year study period (2015–17) with multivariate logistic regression used to identify risk factors for subtherapeutic dosing.

**Results:**

91 adults were treated with azole medication during the study period. A high prevalence of chronic subtherapeutic azole dosing was seen with voriconazole (60.8%) and itraconazole capsule (59.6%) use, representing significant risk factors for subtherapeutic levels. Rapid emergence of azole resistance was additionally seen over the follow-up period with a 21.4% probability of CF patients developing a resistant fungal isolate after 2 years. No significant relationship was found however between subtherapeutic azole dosing and azole resistance emergence.

**Conclusions:**

Our study demonstrates a high prevalence of subtherapeutic azole levels in CF adults with increased risk using itraconazole capsules and voriconazole therapy. We show rapid emergence of azole resistance highlighting the need for effective antifungal stewardship. Further large longitudinal studies are needed to understand the effects of antifungal resistance on outcome in CF and the implications of subtherapeutic dosing on resistance evolution.

## Introduction

Cystic fibrosis (CF) is the most common, life-limiting autosomal recessive genetic disorder in the world.^1^ It is most prevalent in the Caucasian populations of Europe and North America wherein it affects 1 in 2400 live births.^2^ CF is caused by deleterious mutations in the gene that encodes the CF transmembrane conductance regulator (CFTR) protein. To date, over 1500 such mutations have been identified. Dysfunction of the CFTR protein leads to abnormal anion transport and mucociliary clearance, resulting in a multisystem disorder characterized by chronic sinopulmonary infection, and pancreatic insufficiency.^3^ Ineffective clearance of inhaled pathogens from the airways leads to a cycle of infection and progressive inflammation, ultimately culminating in bronchiectasis and respiratory failure.^4^ Over 90% of mortality in CF relates to pulmonary infection,^5^ highlighting the clinical need for efficacious therapy.

There has been extensive research into the contributing role of bacterial pathogens, such as *Staphylococcus aureus* and *Pseudomonas aeruginosa*, in CF-associated lung dysfunction.^6^^,^^7^ However, in concordance with recent findings, the contemporary paradigm is shifting to appreciate the role of fungal organisms, such as *Aspergillus fumigatus* and other filamentous fungi, as an aetiological factor in the progressive respiratory decline associated with CF.^8^ A recent national CF registry data surveillance study suggests *Aspergillus* spp. respiratory tract colonization occurs in ∼30% of individuals with CF.^9^ Although variability exists (likely related to sampling, culture and environmental differences) similar findings have been described in German (37.7%), Argentinian (90%), and Iranian studies, highlighting the global prevalence of *Aspergillus* spp. colonization in CF.^10–12^*A. fumigatus* can cause a wide degree of pathologies, ranging from allergy and sensitization through to invasive aspergillosis, depending on the host immune response.^13^ Within CF, there is likewise a spectrum of *Aspergillus* spp. pulmonary disease manifestations. A previous latent class analysis of a large UK cohort of CF patients presenting with *Aspergillus* spp. infection allowed for their demarcation into three distinct clinical groups, allergic bronchopulmonary aspergillosis (ABPA), *Aspergillus* sensitization, and *Aspergillus* bronchitis.^14^ A number of studies have been performed to understand the clinical implications of *A. fumigatus* colonization in patients with CF. Studies in paediatric CF patient cohorts have shown that those infected or colonized with *A. fumigatus* experienced more pulmonary exacerbations requiring hospital admission as well as greater lung function decline.^15^^,^^16^ A recent study including adults with CF additionally demonstrated a significant relationship between *A. fumigatus* sputum culture positivity and respiratory dysfunction.^17^*Aspergillus* colonization has also been well-described as a risk factor in post-lung transplant mortality.^18^

Currently, triazoles are routinely utilized as a first-line antifungal therapy for the treatment of CF patients with fungal infection and allergy.^18^ Owing to high pharmacokinetic variability, however, regular therapeutic drug monitoring (TDM) is required to optimize dosage to maximize therapeutic potential while minimizing adverse reactions.^19^ Within invasive disease, TDM has been shown to be critical to achieve optimal clinical outcome,^20^ but there is little data within CF-related fungal disease.^21^ TDM can be challenging to perform regularly, as it requires specialist timed blood testing often performed at tertiary care centres with access to specialist equipment. Within a relatively young, independent, working population this represents significant challenges, with added complexity currently within a vulnerable population shielded due to COVID-19 pandemic.

Within CF, limited pharmacokinetic studies have demonstrated high inter-subject variability.^22^ There is to date, however, to our knowledge no ‘real-world’ data on CF azole TDM and few studies analysing associated risk factors for subtherapeutic dosing. This information is highly pertinent given the high (∼30%) prevalence of fungal colonization within the CF patient population,^9^ in conjunction with a lack of consensus between practitioners regarding the most efficacious therapeutic regimens. A recent survey in the UK showed itraconazole was the most frequently prescribed first-line azole medication.^18^ In contrast, however, international consensus statements regarding invasive fungal infection recommend voriconazole as first-line azole therapy, and this is often extrapolated to CF fungal infection.^23^

The importance of TDM is also highlighted by the global emergence of antifungal resistance. We have recently reported an ∼16% prevalence of azole resistance in *A. fumigatus* isolates from a cohort of CF adults.^24^ Specifically, ∼40% of azole-resistant *A. fumigatus* isolates contained the environmentally occurring TR34/L98H allele within the *cyp51A* gene, which has been characterized as a predominant mechanism for triazole resistance.^25^ The implications in CF fungal disease are unclear, but pan-azole resistance has been clearly linked to treatment failure within invasive and non-CF chronic pulmonary aspergillosis.^26^ Fungal exposure to subtherapeutic azole levels over prolonged periods within the context of CF, as a potential risk factor in the development of azole resistance, has not been investigated.

In this study, we present a retrospective adult CF cohort study to analyse real-world azole TDM and risk factors associated with subtherapeutic levels. We further review the development of azole resistance over a 2 year follow-up period, and analyse potential risk factors including type of CF fungal disease and association with subtherapeutic azole levels.

## Patients and methods

### Ethics

Retrospective electronic health record data collection and protocols were approved by the UK Research Ethics Committee (REC reference: 18/HRA/1074).

### Study design

We performed a retrospective longitudinal analysis of all adults in the adult CF centre at the Royal Brompton and Harefield National Health Service (NHS) Foundation Trust on azole therapy between 2015–17. Electronic data records were reviewed for patient demographics, indication, start date of use, prescribed doses and changes, TDM frequency, and use of proton pump inhibitors/antibiotics. CF fungal disease was classified as per modified criteria from Baxter *et al*.^14^: Allergic bronchopulmonary aspergillosis (ABPA): [elevated Total IgE (>1000 kIU/L) and *A. fumigatus-*specific IgE ± *A. fumigatus* culture]; *Aspergillus* spp. sensitization: [elevated Total IgE (but <1000 kIU/L) and *A. fumigatus*-specific IgE but negative *A. fumigatus* culture and negative *A. fumigatus-*specific IgG]; *Aspergillus* bronchitis: (elevated *A. fumigatus-*specific IgG and positive *A. fumigatus* culture with normal Total and *Aspergillus-*specific IgE). Treatment for chronic *Aspergillus* colonization was defined if the patient had ≥2 positive cultures within a 6 month duration over the follow-up period with normal serological tests (IgE and IgG). Non-*Aspergillus* mould colonization was again defined as ≥2 positive cultures within a 6 month duration over the follow-up period. Treatment for mycetoma was based on clinical records and radiographic confirmation. During each clinical visit, the patient’s lung function, BMI and microbiology results were recorded. Azole dosing and TDM interpretation was used and defined as per British Society for Medical Mycology recommendations.^20^ itraconazole target therapeutic trough concentration was >0.5 mg/L, voriconazole/posaconazole were >1 mg/L. Chronic subtherapeutic azole levels in patients were characterized as those who experienced subtherapeutic levels on at least two TDM visits, at least 2 months apart over the duration of the follow-up period. During the follow-up period, new growths of fungal isolates routinely had antifungal susceptibility testing analysis performed, with repeat isolates having annual surveillance unless specifically asked for earlier by the clinical team. Azole-resistant isolates were confirmed with a standard microbroth dilution method according to 2015 EUCAST reference guidelines.^27^ In brief, within the context of *A. fumigatus*, antifungal susceptible (S) was defined as a MIC breakpoint of S ≤ 1 mg/L for itraconazole and posaconazole, and S ≤ 0.12 mg/L for voriconazole. Antifungal resistant (R) was defined as R > 2 mg/L for itraconazole and posaconazole, and R > 0.25 mg/L for voriconazole. Intermediate (I) resistance was defined as any value between S and R. For isolates where EUCAST guideline-based MIC breakpoints were not available (e.g. non-*Aspergillus* species), MIC breakpoints were based on epidemiological cut-off values.

### Statistical analysis

A generalized linear mixed model was fitted to study risk factors associated with subtherapeutic levels. The risk factors studied were age, sex, proton pump inhibitor use, antifungal azole, intravenous antifungals, forced expiratory volume in 1 second (FEV1) percentage, forced vital capacity (FVC), and pancreatic insufficiency. Initially, univariate generalized linear mixed models were fitted to select variables (*P* values <0.2 within univariate analysis) for a multivariate model.

Probability of developing a fungal infection was analysed using a Kaplan–Meier plot with a univariate Cox model with type of CF fungal disease as covariate and time to resistance as outcome. To analyse the time to azole resistance between patients with subtherapeutic azole dosing compared with therapeutic dosing, a multi-state model with 4 stages was developed to reflect the culture results during the follow-up period across the cohort and to study the progression across time: Stage 1: no microbiological growth; Stage 2: fungal isolate grown but no susceptibility testing performed; Stage 3: azole-susceptible fungal isolate; Stage 4: azole-resistant fungal isolate. The patient can progress from stages 1 to 3 and go back to other stages, with stage 4 (azole-resistant fungal isolate) a terminal stage. A logistic model, using only the observations where a fungal microbiology was positive, was fitted including the azole medication and the dose to investigate whether the azole level was relevant for the development of resistance to the medication.

## Results

### Patient demographics

A total of 91 adults with CF were treated with azole medication in the study period (from a total adult CF centre cohort size of 540) and were included in analysis (48 male; 43 female). Between 2015 and 2017, 1275 clinical encounters were recorded; the median number for each individual patient was 22.5 (IQR 18–27) with an average of 7.05 encounters per year per person. Baseline patient demographics are summarized in Table 1.

**Table 1.** Study cohort demographics

Characteristic	Value
Age, years, mean±SD	28.0 ± 8.87
Female, *n* (%)	43 (47.3)
FEV1% predicted, mean±SD	51.1 ± 20.3
PPI use, *n* (%)	56 (61.5)
Pancreatic insufficiency, *n* (%)	83 (91.2)
Reason for antifungal, *n* (%)	
Allergic bronchopulmonary aspergillosis (ABPA)	46 (50.5)
* Aspergillus* bronchitis	21 (23.1)
* Aspergillus* sensitization	6 (6.60)
Chronic *Aspergillus* colonization	4 (4.40)
Mycetoma	5 (5.50)
Non-*Aspergillus* mould growth	9 (9.90)

### Risk factors associated with subtherapeutic levels

Forty-nine patients studied (53.8%) were identified as having chronic subtherapeutic azole levels during the follow-up period based on pre-existing definitions (two or more subtherapeutic TDM results greater than 2 months apart during the follow up period). A general linear mixed model was fitted to determine the risk factors associated with chronic subtherapeutic trough azole levels (Table 2). Two factors were identified as significant risk factors in TDM subtherapeutic outcomes; age (*P* value: 0.031), with younger age being associated with a greater risk, and the type of antifungal azole prescribed (*P* value: 0.024).

**Table 2.** Univariate analysis of risk factors associated with TDM subtherapeutic outcomes

Characteristic	Subtherapeutic levels^a^	Therapeutic levels	*P* value
Total *n* (%)	49 (53.8)	42 (46.2)	**-**
Age, years, mean±SD	24.8 ± 6.38	31.6 ± 9.96	**0.031**
Female, *n*/*N* (%)	26/49 (53.0)	17/42 (40.0)	0.354
BMI	20.4 ± 3.23	21.9 ± 3.80	0.471
FEV1% predicted, mean±SD	50.7 ± 20.3	47.4 ± 3.12	0.135
Proton pump inhibitor use, *n*/*N* (%)	34/49 (69.3)	22/42 (44.9)	0.832
Pancreatic insufficiency, *n*/*N* (%)	45/49 (91.8)	38/42 (90.5)	0.878
Type of antifungal azole prescribed^b^	–	–	**0.024**

*P* values considered significant are shown in bold.

a Defined as at least two subtherapeutic azole levels at least 2 months apart.

b Breakdown for each azole detailed in Table 3.

On multivariate analysis employing risk factor variables with a univariate analysis *P* value <0.2, the type of azole medication was identified as the only significant risk factor. We proceeded to perform a more-detailed analysis of the relationship between azole medication type and subtherapeutic azole levels.

The total time on azole medication, including azole taken prior to study commencement, ranged from 88 to 3287 days (mean value: 1013); 22 patients continued treatment for the full 2 year study period. Over half of patients were prescribed itraconazole; further details on azole administration are shown in Table 3.

**Table 3.** Subtherapeutic levels by triazole medication

Drug	Total TDM visits in follow-up	Subtherapeutic levels, *n* (%)	Odds ratio^a^	95% CI
Itraconazole liquid	62	30 (48.3)	–	–
Itraconazole capsule	57	34 (59.6)	1.58	0.76–3.26
Voriconazole	51	31 (60.8)	1.65	0.78–3.5
Posaconazole	150	72 (48.0)	0.98	0.54–1.78
Isavuconazole	3	0 (0)	NA	NA

NA, not applicable.

a Odds ratio for subtherapeutic azole TDM for individual triazole drug compared with itraconazole liquid.

**Figure 1.**
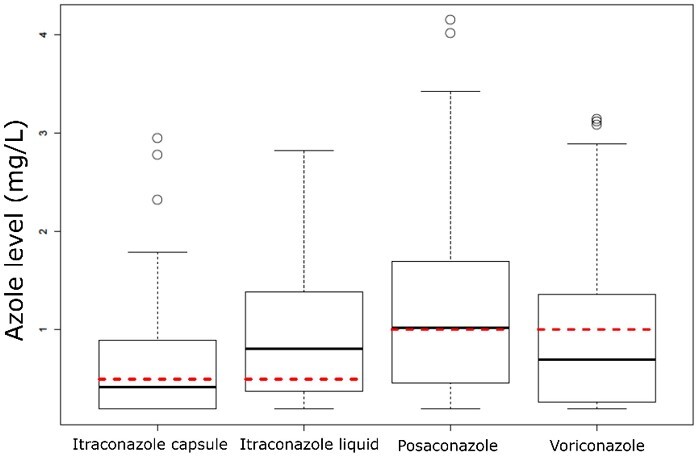
Therapeutic drug levels of individual triazole therapy over study duration. Red dashed line represents target therapeutic trough level for each azole medication.

Figure 1 shows the trough azole levels of individual medications across the TDM follow-up period. The median trough azole levels for itraconazole capsules and voriconazole (Figure 1) were below the target trough concentration (0.5 mg/L and 1 mg/L, respectively), with more than 50% of itraconazole capsule (59.6%) and voriconazole (60.8%) exhibiting subtherapeutic levels. Voriconazole and itraconazole capsules were 1.378 times more likely to have subtherapeutic levels compared with posaconazole [delayed-release (M/R) formulation] and liquid formulation itraconazole. However, although better, itraconazole liquid trough levels were still subtherapeutic in 48.3% of tests with posaconazole M/R levels subtherapeutic in 48% (Table 2).

### Azole resistance evolution

**Figure 2.**
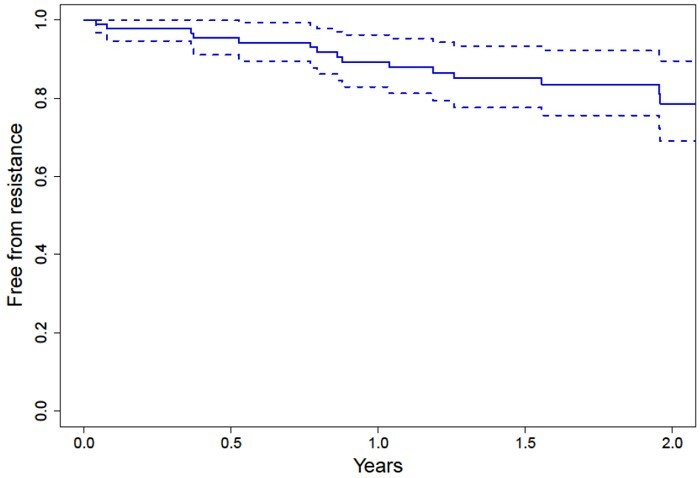
Kaplan–Meier probability of freedom from azole resistance of pathogenic mould isolates over study duration.

We next investigated the emergence of new azole resistance across all isolated pathogenic moulds (including *A. fumigatus* and non-*Aspergillus* spp.), over a 2 year period. At study commencement, 88 participants had positive fungal cultures for a pathogenic mould and were therefore included in this analysis. At time of inclusion, only two patients had pre-existing azole resistance (one patient with *A. fumigatus* and another patient with *Rasamsonia argillacea* colonization). The probability of a patient developing azole antifungal resistance over the study duration was initially investigated with a Kaplan–Meier plot (Figure 2). 10.7% (3.8%–17.1%) of patients had azole-resistant fungal infection after 1 year and 21.4% (11.5%–41%) after 2 years (Figure 2).

**Figure 3.**
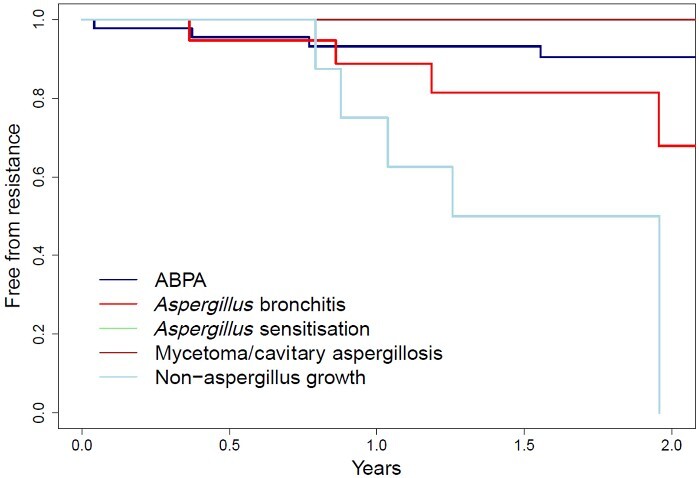
Kaplan–Meier probability of freedom from azole resistance or pathogenic mould isolates over study duration with underlying CF fungal disease categories.

We performed further analysis to see whether the underlying CF fungal disease subtype affected the development of antifungal resistance. A univariate Cox model with the reason for antifungal azole use as covariate and the time to resistance as outcome was fitted. Although subgroup analysis was limited by the total number of patients, this revealed a significant relationship, with a *P* value of 0.0034 (Figure 3). Significance was driven by increased azole resistance seen in patients treated for non-*Aspergillus* moulds [only 75% (50.3%–100%)] of patients free from resistance at year 1, with *Aspergillus* bronchitis showing a non-significant trend towards greater acquisition of azole resistance (Figure 3 and Table 4).

**Table 4.** Freedom from resistance after 2 years in CF fungal disease subtypes requiring azole therapy

Year	Number at risk	Number of events	Survival^a^
Allergic bronchopulmonary aspergillosis			
0	46	0	1 (1–1)
1	39	3	0.932 (0.861–1)
2	21	1	0.905 (0.82–0.999)
*Aspergillus* bronchitis			
0	19	0	1 (1–1)
1	15	2	0.888 (0.753–1)
2	5	2	0.678 (0.442–1)
*Aspergillus* sensitization			
0	6	0	1 (1–1)
1	5	0	1 (1–1)
2	1	0	1 (1–1)
Mycetoma/cavitary aspergillosis			
0	4	0	1 (1–1)
1	4	0	1 (1–1)
2	3	0	1 (1–1)
Non-*Aspergillus* spp. growth			
0	9	0	1 (1–1)
1	6	2	0.75 (0.503–1)

a Freedom from resistance (95% CI).

We next looked at the prevalence of azole resistance in individual fungal species over the duration of the follow-up period. 1618 sputum samples were collected and 628 were positive for fungal colonization; 296 for *Candida* spp. (excluded from further analysis) and 332 for other fungi, see Table 5. Of these, a total of 276 samples were measured for azole resistance based on the MIC recorded. 124 were azole resistant (44.9%). Of the resistant isolates, 41.9% were *A. fumigatus*. We further looked at the emergence of azole resistance of different fungal species, within our CF cohort, on a year-by-year basis (Table 5). This showed the evolution of azole resistance in *A. fumigatus*, with 24.1% of analysed isolates azole resistant in the first year of the follow-up period and a subsequent increase in the second year to 45.8% (Table 5).

**Table 5.** Azole resistance in analysed fungal isolates

Fungal species	Positive isolates from culture *N* (% of total isolates)	Azole-resistant isolates^a^*N* (%)^b^		
		Total	Year 1	Year 2
*Aspergillus fumigatus*	170 (51.2)	52 (38.0)	17 (23.0)	35 (55.6)
*Aspergillus* spp. (non-*fumigatus*)	2 (0.60)	1 (50.0)	1 (50.0)	–
*Scedosporium apiospermum*	88 (26.5)	6 (9.0)	3 (14.3)	3 (7.0)
*Scedosporium prolificans* ^c^	4 (1.20)	4 (100)	4 (100)	–
*Exophiala dermatitidis*	45 (13.6)	12 (26.1)	12 (44.0)	0 (0)
*Rasamsonia* spp.	22 (6.33)	12 (75.0)	2 (33.3)	10 (100)
Other	1 (0.30)	1 (100)	1 (100)	–

a Included only fungal isolates in which susceptibility assays were performed.

b Percentage of azole-resistant isolates of the total isolates within the year analysed.

c Intrinsically azole resistant.

We further analysed evolution of *A. fumigatus* specific azole resistance over the duration of the follow-up period (Table 6). 77% of tested *A. fumigatus* isolates exhibited susceptibility to itraconazole during the first year of the follow up period. However, by the second year, the prevalence of itraconazole resistance in tested isolates rose to over 50%. There was an additional trend towards increased voriconazole and posaconazole resistance, with increased intermediate resistance in year 2.

**Table 6.** Breakdown of resistance by drug for *A. fumigatus* (i.e. all isolates where susceptibility testing was done)

	Azole-resistant isolates^a^*n* (%)	
Drug/classification	Year 1	Year 2
Itraconazole		
Susceptible	57 (77.0)	24 (39.3)
Intermediate	2 (2.7)	6 (9.8)
Resistant	15 (20.3)	33 (54.1)
Voriconazole		
Susceptible	59 (78.7)	36 (59.0)
Intermediate	16 (21.3)	23 (37.7)
Resistant	0 (0)	2 (3.3)
Posaconazole		
Susceptible	1 (33.3)	4 (18.18)
Intermediate	2 (66.6)	16 (72.7)
Resistant	0 (0)	2 (9.09)

a Included only fungal isolates in which susceptibility assays were performed.

Lastly, we aimed to analyse any effect of subtherapeutic azole levels on development of antimicrobial resistance using a multistate model. None of the previously described covariates including subtherapeutic TDM outcome were significantly independently associated with increased azole resistance. The transition probability matrix indicates that there is a 16% (95% CI: 8.4%–40.1%) probability of a fungal isolate developing azole resistance from a previous state of azole susceptibility (Table 7). The sojourn time represents the average time spent in each stage. Once a patient had grown a susceptible fungal isolate they remained in that state for an average of 49 days (95% CI: 20–102).

**Table 7.** Transition probability matrix using a multistate model

	Probability of transitioning into state:		
State	No isolate or isolate but no susceptibility testing (Stage 1/2)	Azole-susceptible isolate (Stage 3)	Azole-resistant isolate (Stage 4)
No isolate or isolate but no susceptibility testing (Stage 1/2)	0.806 (0.666–0.87)	0.084 (0.045–0.139)	0.11 (0.063–0.244)
Azole-susceptible isolate (Stage 3)	0.761 (0.532–0.834)	0.079 (0.037–0.142)	0.16 (0.084–0.409)

## Discussion

In this study, we present a retrospective ‘real-world’ longitudinal cohort study of adults with CF to investigate the pharmacokinetics of first-line antifungal medication and the evolution of triazole resistance. We highlight the wide variability and subtherapeutic dosing of azole medications for prolonged periods in a CF cohort, analyse potential risk factors and effects on antifungal resistance.

Univariate analysis showed younger patients and specific azole medications were at greater risk of subtherapeutic dosing. Although not significant in multivariate analysis, the finding of increased subtherapeutic dosing in younger patients requires further study. In this retrospective study, we were unable to analyse whether adherence is a factor, however, numerous studies have shown that adherence in CF is often sub-optimal and adult transition from paediatrics can be a contributing factor.^28^^,^^29^ Within multivariate analysis, a significant association with subtherapeutic dosing was only seen with the type of azole medication used. Itraconazole capsules and voriconazole had a significantly increased likelihood of subtherapeutic TDM outcome as compared with itraconazole liquid and posaconazole, with median TDM values below target therapeutic trough concentrations. These findings are consistent with the known poor bioavailability of itraconazole capsules and variable pharmacokinetics of itraconazole and voriconazole.^30^^,^^31^ This raises significant concerns regarding extrapolating international invasive fungal infection guideline-based recommendations where voriconazole is considered first-line therapy for CF fungal disease, alongside the significant proportion of responders in a recent UK survey concerning treatment of CF fungal disease who advocate itraconazole capsules as first line therapy.^18^ Itraconazole liquid is known to have better bioavailability compared with capsules, but its taste and lack of ease of administration can potentially affect compliance.^32^ Delayed-release posaconazole which was used in this study has been shown to have enhanced bioavailability and more consistent pharmacokinetics, with recent papers suggesting therapeutic levels are achievable in the majority of non-CF chronic aspergillosis cases at standard doses.^33^ Although better when compared with itraconazole capsules and voriconazole, our study still showed a significant number of subtherapeutic posaconazole levels.

Within our cohort, a small number of patients were prescribed isavuconazole, a novel azole antifungal with very good bioavailability, predicable pharmacokinetics and fewer drug–drug interactions.^34^ In this small number, all trough levels were within a likely therapeutic range (deemed 1–5 mg/L) but further studies are required to determine the need for regular TDM of isavuconazole in CF.

Several previous studies have reported the emergence of multi-azole-resistant *A. fumigatus* isolates derived from CF patients.^27^^,^^35^^,^^36^ Our study findings provide a timeline for the development of azole resistance. Multiple studies have shown the most common species responsible for fungal infections in CF patients include *A. fumigatus*, *Scedosporium apiospermum*, and *Exophiala dermatitidis*.^37^ Our data support these previous findings, with these species comprising 91.8% of all isolated fungi, and 84.6% of the azole-resistant isolates. Consistent with the literature, we found the most prevalent fungus among our isolates was *A. fumigatus*, making up 51.2% of the sample. We showed significant evolution of azole resistance over a 2 year period, with 41.9% overall of *A. fumigatus* isolates having azole resistance. Over the 2 year follow-up duration, significant emergence of itraconazole resistance was seen, with increased intermediate resistance to voriconazole and posaconazole. The rapid emergence of azole resistance seen over a relatively short duration (2 years) potentially highlights the need for more frequent antimicrobial susceptibility testing in high-risk cohorts such as CF.

Within our study period, we demonstrate a considerable risk of the emergence of antifungal resistance in adults with CF, with ∼20% of study subjects having azole-resistant fungal isolates. Although limited by the small numbers in our cohort study, the underlying CF fungal disease sub-type appears to influence azole resistance in our cohort. Intriguingly, azole resistance development was highest during treatment for non-*Aspergillus* mould infections. There is currently no genetic understanding of azole resistance in non-*Aspergillus* filamentous fungi, with the clinical implications of non-*Aspergillus* azole resistance again uncertain. A number of studies have indicated, however, that colonization with *Scedosporium* spp. and *Exophiala* spp. can be detrimental in certain patients and are a risk factor in post-lung transplantation mortality, although their pathogenic role in driving CF disease progression is less-well-described in comparison with *A. fumigatus*.^38–40^ Our findings demonstrate the need for further surveillance to monitor the emergence, molecular mechanism and clinical implications of antifungal drug resistance in non-*Aspergillus* pathogenic moulds in CF. Our results suggest a trend towards increased azole resistance development in *Aspergillus* bronchitis compared with ABPA, however, this requires confirmation in larger studies. The increased tracheobronchial burden seen in *Aspergillus* bronchitis presents a plausible mechanistic basis given the tendency to form biofilms, reducing mould exposure to antifungal drugs and creating a hypoxic microenvironment putatively encouraging the emergence and propagation of azole resistance.^41^^,^^42^

Our study has a number of limitations. Although we demonstrate the significant variability and high prevalence of subtherapeutic azole dosing in CF, we were unable to analyse the potential impact of cytochrome P450 genetic polymorphisms on azole metabolism.^43^ Larger studies are also necessarily to fully evaluate the impact of drug–drug interactions on therapeutic dosing. A further limitation of our study is the uncertainty regarding the clinical implications of antifungal resistance in our cohort. In our cohort, there was a small number of cases infected with pan-azole-resistant *A. fumigatus*. These cases and outcomes are briefly summarized in Table S1 (available as Supplementary data at *JAC-AMR* Online) and highlight the variability in outcome in CF fungal disease with two out of three cases clearly showing disease progression, but one showing stability following regular intravenous antifungal salvage therapy. Further larger multicentre longitudinal observational studies are required to fully determine the significance of azole resistance evolution for pathogen virulence, persistence, host–pathogen interaction and clinical outcome. A number of studies, however, have demonstrated the association of pan-azole-resistant *A. fumigatus* with poor clinical outcome and increased risk of mortality post lung transplantation, providing some evidence for the clinical significance of our findings.^15–17^^,^^44^ Further studies are ongoing to determine the molecular basis of azole resistance acquisition and evolution in this cohort. Overall, however, our findings illustrate the escalating emergence and propagation of widespread azole resistance within the fungal species most associated with human diseases such as CF, which may, in time, and without intervention, come to pose a major global health hazard.

There is little data pertaining to the relationship between subtherapeutic azole levels and the emergence of antifungal resistance. Contrary to non-CF cohorts, we show significant prevalence of subtherapeutic TDM despite use of newer azole drugs such as posaconazole, with supposedly better pharmacokinetic properties, in cohorts with high prevalence of azole-resistant *A. fumigatus*, such as CF. In our study, we were unable to show an association between subtherapeutic azole levels and the development of azole resistance. In addition, the study size did not allow for an in-depth analysis of the impact of time of azole exposure on resistance development. Further large prospective longitudinal studies alongside molecular characterization of resistance are likely necessary to fully understand the impact of long-term subtherapeutic azole exposure in driving resistance in CF.

Our study highlights the need for regular TDM and the importance of antifungal stewardship in CF across all widely used azole drugs. Although antimicrobial stewardship (including antifungal stewardship) has been increasingly taken up within an invasive fungal disease (IFD) setting, there is limited data and reports of its integration and impact within a chronic pulmonary fungal disease setting such as seen in CF, with recent international consensus documents focusing on IFD.^45^^,^^46^ Antifungal stewardship implementation in CF fungal disease poses further challenges with often long duration of treatment, a young, busy, working population making regular timed blood tests at tertiary centres (often long distances away) difficult, plus marked pharmacokinetic variability.^47^ The advent of novel CFTR modulators with resultant drug–drug interactions alongside the need for shielding in a COVID-19 pandemic has only heightened these issues and make integration of robust antifungal stewardship programmes in CF fungal disease a priority.^48^

In summary, our retrospective study of real-world azole TDM in a large adult CF cohort highlights significant subtherapeutic dosing alongside rapid emergence of azole resistance, highlighting the need for integrated antifungal stewardship in CF fungal disease. In contrast to widely adopted practices in CF fungal management and international recommendations, we identify individual azole therapy with itraconazole capsule and voriconazole with an increased risk of subtherapeutic dosing. Further, we show rapid development of *A. fumigatus* azole resistance over a short follow-up duration in adults with CF and significant emergence of azole resistance in non-*Aspergillus* mould infection. Further large multicentre prospective studies are required to elucidate the impact of subtherapeutic azole dosing on the molecular mechanism of azole resistance evolution.

## Funding

This work was supported by a Gilead UK Fellowship in Invasive Fungal Infection (A.S.) and MRC Clinical Academic Research Partnership award (A.S.). A.S. and M.C.F. are supported by the MRC centre grant MRC; MR/R015600/1. M.C.F. is a CIFAR fellow. D.A.-J. is supported by the Department of Health and Social Sciences Centre for Antimicrobial Optimisation and a Cystic Fibrosis Trust Strategic Research Centre programme grant (TrIFIC).

## Transparency declarations

A.S. reports grants from Vertex pharmaceuticals and Gilead Sciences and speaker fees from Pfizer and Gilead Sciences. M.C.F. reports speaker fees from Gilead Sciences. All other authors have none to declare.

## Supplementary data

Table S1 is available as Supplementary data at *JAC-AMR* Online

## Supplementary Material

dlab026_Supplementary_Data
