# Erratum to: A retrospective ‘real-world’ cohort study of azole therapeutic drug monitoring and evolution of antifungal resistance in cystic fibrosis

**DOI:** 10.1093/jacamr/dlab086

**Published:** 2021-07-12

**Authors:** M Di Paolo, L Hewitt, E Nwankwo, M Ni, A Vidal-Diaz, M C Fisher, D Armstrong-James, A Shah

*JAC-Antimicrobial Resistance* 2021; https://doi.org/10.1093/jacamr/dlab026

In the author list, the name ‘E. Nwanko’ should have appeared as ‘E. Nwankwo’. This has now been corrected in the published version of the article.

The authors apologize for this error.

