# Supplementary material for: A retrospective ‘real-world’ cohort study of azole therapeutic drug monitoring and evolution of antifungal resistance in cystic fibrosis
Source: JAC Antimicrob Resist. 2021 Mar 16;3(1):dlab026. doi: 10.1093/jacamr/dlab026 (PMC8210303; doi:10.1093/jacamr/dlab026)
Supplement: dlab026_Supplementary_Data [file dlab026_supplementary_data.docx]

**Supplementary data**

**Table S1.** Case series for patients with azole evolution requiring Isavuconazole

|  | Reason for antifungal | *Aspergillus* resistance split into year | | | Other micro | Previous treatment | Antifungal treatment used during follow up | Outcome |
| --- | --- | --- | --- | --- | --- | --- | --- | --- |
|  |  | 15’ | 16’ | 17’ |  |  |  |  |
| Case 1 | Mycetoma/Cavitatory aspergillosis | x | Itra | Itra/Vori | *Candida* | Itraconazole (liquid), commenced 2009 | Itraconazole stopped in 2016 due to Resistant fungal growth. Started Voriconazole which developed resistance, so changed to Isavuconazole in 2017 | Multiple exacerbations requiring IV antifungal therapy. Lung function decline. |
| Case 2 | Aspergillus bronchitis | x | x | Itra/Vori/Posa | *Candida* | Itraconazole (liquid), commenced 2016 | Itraconazole stopped in due to resistance in Feb 2017; started Voriconazole which developed resistance in April 2017, changed to Posaconazole which had resistance in Dec 2017. | Multiple exacerbations requiring IV antifungal therapy. Lung function relatively stable |
| Case 3 | Aspergillus bronchitis | x | x | Posa | *Scedosporium prolificans, Scedosporium apiospermum* | Posaconazole, commenced 2012 | Posaconazole stopped due to intermediate fungal growth. Isavuconazole started in 2017 | Multiple exacerbations requiring IV antifungal therapy. Lung function decline |
